# Galangin Mitigates Oxidative Damage Induced by Environmental Stresses in Skin Keratinocytes

**DOI:** 10.7150/ijms.112872

**Published:** 2025-07-28

**Authors:** Herath Mudiyanselage Udari Lakmini Herath, Mei Jing Piao, Kyoung Ah Kang, Pincha Devage Sameera Madushan Fernando, Herath Mudiyanselage Maheshika Madhuwanthi Senavirathna, Young Sang Koh, Eui Tae Kim, Suk Ju Cho, Jin Won Hyun

**Affiliations:** 1 College of Medicine, and Jeju Research Center for Natural Medicine, Jeju National University, Jeju 63243, Republic of Korea.; 2 Jeju National University Hospital, College of Medicine, Jeju National University, Jeju 63241, Republic of Korea.

**Keywords:** galangin, environmental stress, reactive oxygen species, apoptosis

## Abstract

Particulate matter 2.5 (PM_2.5_) is a major air contaminant that causes skin damage by interacting with ultraviolet (UV) radiation. Exposure to those environmental stresses leads to oxidative skin damage and apoptosis. Although galangin is a natural flavonoid with antioxidant and several bioactive properties, its antioxidative effects following combined PM_2.5_ and UVB exposure have not been fully investigated. Therefore, the aim of this study was to investigate the protective effect of galangin against PM_2.5_- and UVB-induced oxidative stress and apoptosis in keratinocytes. Human HaCaT keratinocytes were pre-treated with galangin and treated with PM_2.5_ and/or UVB. Intracellular reactive oxygen species (ROS) levels, lipid peroxidation, protein oxidation, DNA damage, mitochondrial damage, apoptotic protein expression, and cellular apoptosis were assessed using flow cytometry, confocal microscopy, and western blotting. Galangin reduced ROS levels, lipid peroxidation, protein oxidation, DNA damage, mitochondrial damage, and cellular apoptosis caused by PM_2.5_ and/or UVB exposure. Additionally, galangin attenuated PM_2.5_- and UVB-induced upregulation of apoptosis-related proteins and restored the expression of anti-apoptotic proteins. PM_2.5_ and/or UVB enhanced cellular apoptosis by activating the mitogen-activated protein kinase (MAPK) signaling pathway. Notably, combined treatment with MAPK inhibitors and galangin demonstrated a protective effect against PM_2.5_- and/or UVB-induced apoptosis. Galangin protected human keratinocytes against PM_2.5_- and/or UVB-induced cellular damage by inhibiting MAPK signaling, suggesting that it may be a beneficial ingredient in skin care products designed to safeguard the skin from the detrimental effects of environmental stress.

## Introduction

Galangin (3,5,7-trihydroxyflavone) is primarily extracted from the roots of *Alpinia officinarum* and *Helichrysum aureonitens* 1, 2. Galangin, commonly found in traditional Chinese medicine, has been scientifically proven to possess anti-cancer, anti-inflammatory, and anti-oxidative properties 3. A previous study demonstrated that galangin inhibited UVB-induced oxidative damage in HaCaT keratinocytes 2. Additionally, galangin protects cells from UVB by enhancing the expression of the antioxidant enzymes glutamate-cysteine ligase catalytic subunit and glutathione synthetase via activation of nuclear factor-erythroid 2-related factor 4.

Airborne pollution is a worldwide health issue linked to increasing rates of respiratory, cardiovascular, skin-related, and neurological diseases, as well as premature deaths 5-9. Particulate matter (PM) is one of the worst types of air pollutants and is a heterogeneous mixture of liquid droplets and small particles composed of metals, organic compounds, and dust or soil particles. PM can be categorized into the following groups based on their size: coarse (diameter 2.5-10 μm, PM_10_), fine (diameter ≤ 2.5 μm, PM_2.5_), and ultrafine (diameter < 100 nm, PM_0.1_) particles 10. Vehicle emissions are among the important sources of polycyclic aromatic hydrocarbons (PAHs) 11. Importantly, PAHs form a complex with aryl hydrocarbon receptor (AhR), which is translocated to the nucleus, triggering the production of reactive oxygen species (ROS) 12.

The skin is the primary target of ultraviolet (UV) radiation, including UVA, UVB, and UVC 13, among which UVB radiation is the harmful and genotoxic form of sunlight 14. UVB irradiation induces excessive ROS production, damages skin integrity, accelerates skin aging, triggers inflammation, causes DNA damage, and induces cell death 15. Additionally, UVB exposure contributes to the development of skin cancer 16.

PM and some PAHs can absorb UV radiation and produce ROS, such as singlet oxygen, which can cause lipid peroxidation, DNA damage, and cell death 17. Boira et al. reported that combined exposure to UVA radiation and PAHs or particulate matter caused a more pronounced phototoxic effect than UV radiation alone, which was attributed to ROS production and mitochondrial dysfunction 18. Although galangin has been shown to possess some beneficial effects, its potential to counteract damage caused by PM_2.5_ and UVB co-exposure has not been fully explored. Considering this knowledge gap, the aim of this study was to investigate the protective effect of galangin against UVB radiation- and PM_2.5_-induced toxicity in HaCaT keratinocytes, with the goal of identifying a potential therapeutic strategy for environmental skin damage.

## Materials and Methods

### PM_2.5_ and sample preparation

PM_2.5_ (Diesel particulate matter, NIST standard reference material 1650b) was purchased from Sigma-Aldrich (St. Louis, MO, USA). A stock solution of PM_2.5_ (25 mg/mL) was prepared in dimethyl sulfoxide (DMSO) and sonicated for 30 min to prevent particle agglomeration. All experiments were performed within 1 h of stock preparation to ensure consistency and avoid fluctuations in PM_2.5_ concentration 19. Galangin was purchased from Santa Cruz Biotechnology (Santa Cruz, CA, USA).

### Cell culture, PM_2.5_ and UVB exposure

HaCaT cells were obtained from the Cell Lines Service (Eppelheim, Germany). The cells were cultured at 37°C in a humidified incubator with 5% CO_2_ and grown in Dulbecco's Modified Eagle's medium supplemented with 10% heat-inactivated fetal bovine serum and 1% antibiotic-antimycotic solution. Thereafter, the cells were treated with PM_2.5_ (50 µg/mL) and exposed to UVB radiation (30 mJ/cm^2^) using a CL-1000M UV Crosslinker (UVP, Upland, CA, USA).

### Cell viability assessment

To assess the cytotoxic effects of PM_2.5_, 3-(4,5-dimethylthiazol-2-yl)-2,5-diphenyltetrazolium bromide (MTT; Sigma-Aldrich) and trypan blue assays were conducted. Briefly, cells were treated with 40 μM of galangin, MAPK inhibitors (U0126, SB203580, SP600125; Sigma-Aldrich), and PM_2.5_ (50 µg/mL) for 24 h. Thereafter, 2 mg/mL MTT was applied to the cells and incubated for 4 h to generate formazan crystals. After dissolving these crystals in DMSO, the absorbance at 540 nm was recorded using a microplate reader (Molecular Devices, San Jose, CA, USA). For the trypan blue assay, cells were stained with 5 µL of 0.1% trypan blue solution and incubated for 5 min at 20°C. Finally, viable and dead cells were counted under a light microscope at ×20 magnification, and the percentage of viable cells was calculated as unstained cells/(unstained cells + stained cells) × 100% 19.

### Cellular ROS assessment

Intracellular ROS levels were determined using 2′,7′-dichlorodihydrofluorescein diacetate (H_2_DCFDA; Molecular Probes, Eugene, OR, USA). Briefly, seeded cells were treated with galangin (40 μM), N-acetyl cysteine (NAC, 1 mM; Sigma-Aldrich), PM_2.5_ (50 µg/mL), and UVB (30 mJ/cm^2^). After staining the cells with H_2_DCFDA, data were obtained using a flow cytometer (Becton Dickinson, Franklin Lakes, NJ, USA) and a confocal microscope (Olympus, Tokyo, Japan).

### Lipid peroxidation assay

Lipid peroxidation was measured using diphenyl-1-pyrenylphosphine (DPPP) fluorescent probe (Molecular Probes; Eugene, OR, USA). After treatment with galangin, PM_2.5_, and UVB, 5 μM of DPPP was added to stain the cells. Fluorescence images were obtained using a confocal microscope (Olympus).

### Protein carbonylation assay

Protein carbonylation was quantified using a protein carbonyl ELISA kit (OxiSelect™, Cell Biolabs, San Diego, CA, USA), according to the manufacturer's instructions.

### Detection of 8-oxoguanine (8-oxoG)

Cells were stained with an avidin-TRITC conjugate (Sigma-Aldrich) and fixed on a chamber slide. Fluorescence images were acquired via a confocal microscope (Olympus).

### Ca^2+^ level quantification

Cells were seeded into chamber slides, treated with 40 μΜ of galangin for 30 min, and exposed to 50 μg/mL of PM_2.5_ for another 24 h. Thereafter, the treated cells were stained with Fluo-4 acetoxymethyl ester (Fluo-4 AM) (Thermo Fisher Scientific). Finally, fluorescence images were obtained using a confocal microscope (Olympus).

### Mitochondrial membrane potential (Δψ_m_) analysis

Cells were incubated with 5,5′,6,6′-tetrachloro-1,1′,3,3′ tetraethylbenzimidazolylcarbocyanine iodide (JC-1) dye (Thermo Fisher Scientific, Waltham, MA, USA), and fluorescence images were obtained using a confocal microscope (Olympus).

### Nuclear staining with Hoechst 33342

Cells were incubated with Hoechst 33342 (a DNA-specific fluorescent dye; Thermo Fisher Scientific) for 10 min. Thereafter, the level of nuclear condensation in stained cells was assessed using a fluorescence microscope and CoolSNAP-Pro color digital camera (Media Cybernetics, Rockville, MD, USA).

### Western blot analysis

Protein (30 μg) was separated using 10% SDS-polyacrylamide gel electrophoresis, transferred onto a nitrocellulose membrane, and blocked with 3% bovine serum albumin at 20°C for 1 h 20. After blocking, the membranes were incubated with primary antibodies against phospho-histone H2A variant X (phospho-H2A.X), myeloid cell leukemia-1 (Mcl-1), caspase-3, extracellular signal-regulated kinase (ERK), phospho-ERK, c-Jun N-terminal kinase (JNK), phospho-JNK, mitogen-activated protein kinase kinase (MEK1/2), phospho-MEK1/2, stress activated protein kinase (SAPK)/ERK kinase-1 (SEK1), phospho-SEK1, which were supplied from Cell Signaling Technology (Danvers, MA, USA); B-cell lymphoma 2 (Bcl-2), Bcl-2-like protein 11 (Bim), Bcl-2-associated X protein (Bax), actin, caspase-9, poly (ADP-ribose) polymerase (PARP), p38, and phospho-p38, which were supplied from Santa Cruz Biotechnology (Dallas, TX, USA). Thereafter, anti-IgG secondary antibodies were added to the membranes for incubation (Thermo Fisher Scientific). Protein bands were detected using an enhanced chemiluminescence western blot detection kit (Amersham, Little Chalfont, Buckinghamshire, UK).

### Statistical analysis

All data are presented as the mean ± standard error of the mean. Significant differences between groups were determined using analysis of variance and Tukey's post hoc test. Statistical significance was set at *p* < 0.05.

## Results

### Galangin reduced PM_2.5_-enhanced cellular ROS

**Figure 1:**
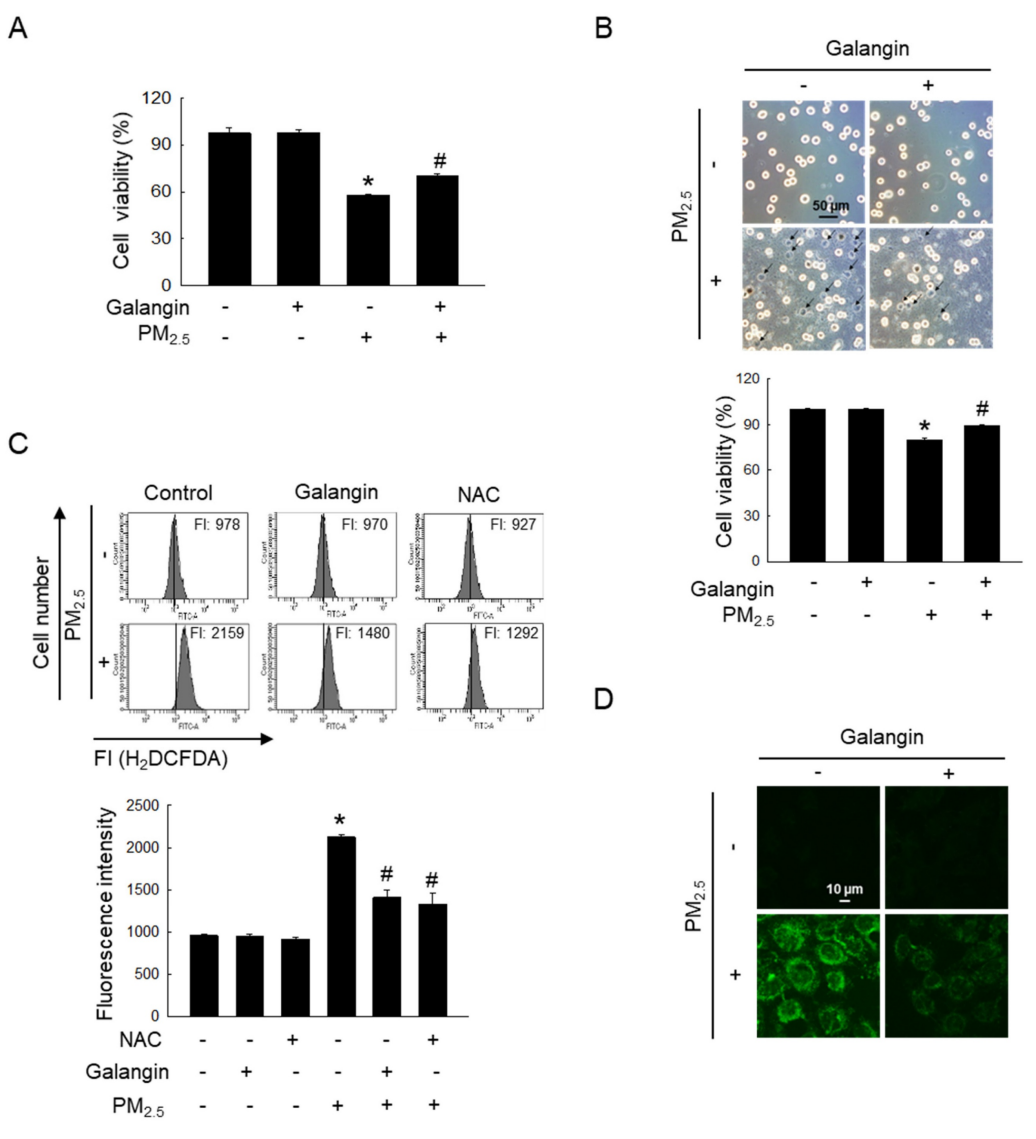
Protective effect of galangin against PM_2.5_-induced cytotoxic effects and ROS generation. HaCaT cells were pretreated with 40 µM of galangin, followed by exposure to PM_2.5_ (50 µg/mL). (A, B) Cell viability was evaluated using (A) MTT assay and (B) trypan blue stain. (C, D) Intracellular ROS levels were evaluated using (C) flow cytometry and (D) confocal microscopy following staining with H_2_DCFDA. (A-C) *p < 0.05, ^#^*p* < 0.05 versus control and PM_2.5_. PM_2.5_, particulate matter 2.5; MTT, 3-(4, 5-dimethylthiazol-2-yl)-2,5-diphenyltetrazolium bromide; ROS, reactive oxygen species; H_2_DCFDA, 2′,7′-dichlorodihydrofluorescein diacetate.

Our previous study demonstrated that galangin exhibited significant DPPH radical-scavenging activity at concentrations between 20-100 μM, comparable to the antioxidant effect of 1 mM NAC 2. Among the concentrations examined, 40 μM was identified as the optimal concentration for ROS scavenging in HaCaT keratinocytes. Based on these findings, subsequent experiments in this study were conducted using 40 μM galangin. MTT assay showed that galangin significantly ameliorated PM_2.5_-induced decrease in HaCaT cell viability (*p* < 0.05; Figure 1A). Notably, trypan blue assay showed that the galangin treatment in the PM_2.5_ group had a lower trypan blue-stained cell population than the PM_2.5_ group, confirming the protective effect of galangin (*p* < 0.05; Figure 1B). Additionally, we investigated the protective effect of galangin against PM_2.5_-induced intracellular ROS production. H_2_DCFDA assay showed that galangin inhibited PM_2.5_-induced ROS production, showing a similar inhibitory effect as NAC, a well-known antioxidant (*p* < 0.05; Figures 1C and 1D).

### Galangin protected cellular components against PM_2.5_-induced oxidative damage

**Figure 2:**
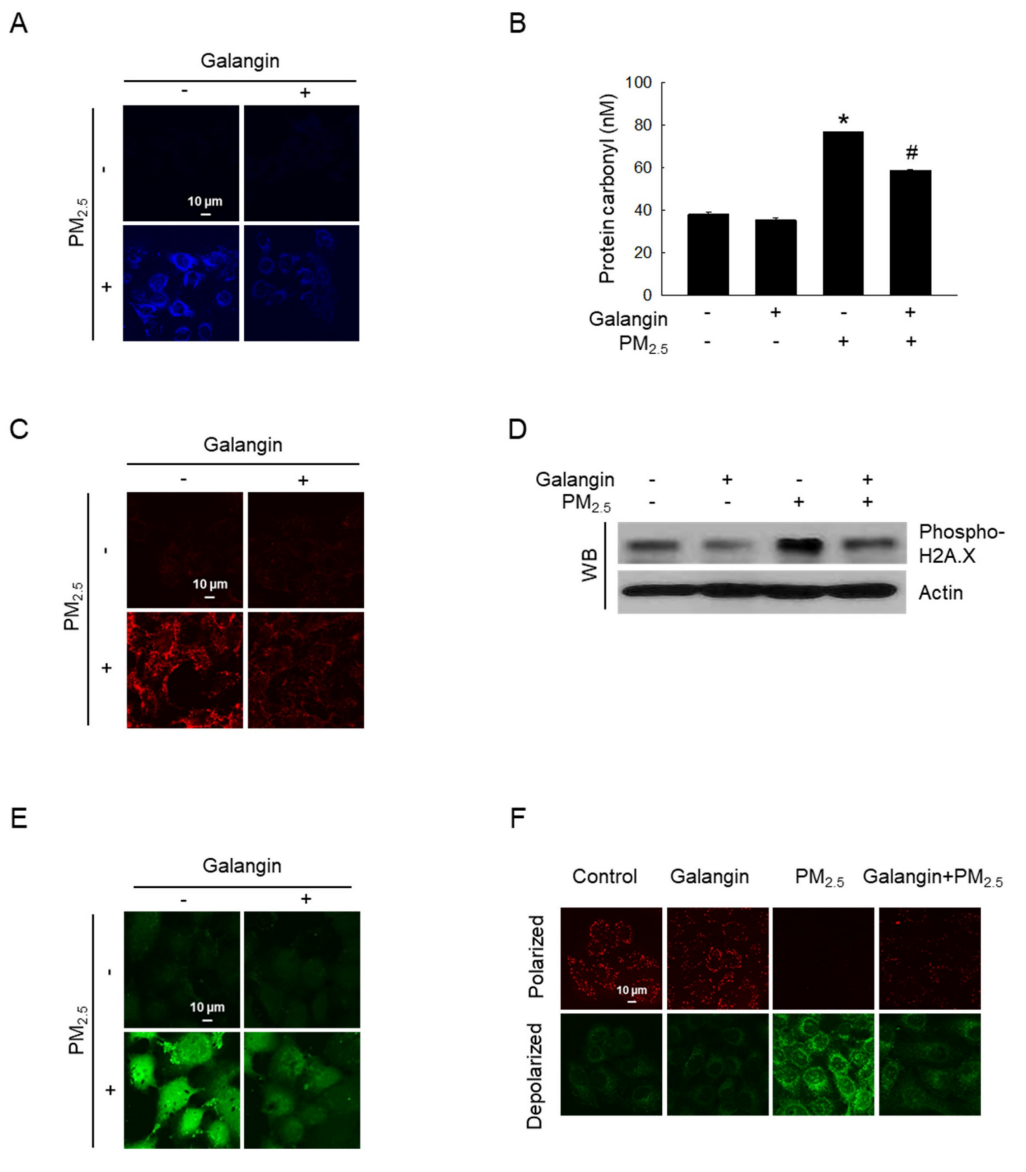
Protective effect of galangin against PM_2.5_-induced macromolecular damage. (A) DPPP staining confirmed lipid peroxidation. (B) Protein oxidation was confirmed by assessing carbonyl production. *p < 0.05, ^#^*p* < 0.05 versus control and PM_2.5_. (C) 8-OxoG level after an avidin-TRITC conjugate staining was detected using confocal microscopy. (D) phospho-H2A.X protein expression was analyzed using western blot analysis, with actin as the loading control. (E) Intracellular Ca^2+^ level was analyzed using Fluo-4 AM staining using confocal microscopy. (F) Mitochondrial membrane potential (Δψ_m_) was examined using JC-1 staining. PM_2.5_, particulate matter 2.5; DPPP, diphenyl-1-pyrenylphosphine; JC-1, 5,5′,6,6′-tetrachloro-1,1′,3,3′ tetraethylbenzimidazolylcarbocyanine iodide.

Macromolecular damage was evaluated to determine whether increased oxidative stress causes damage to cells. PM_2.5_-induced lipid oxidation, which was ameliorated by galangin treatment (Figure 2A). Additionally, galangin significantly inhibited PM_2.5_-induced protein carbonylation (an oxidative protein modification) in HaCaT cells (*p* < 0.05; Figure 2B). Moreover, galangin decreased 8-oxoG level in PM_2.5_-exposed cells compared with that in untreated cells (Figure 2C). Importantly, galangin downregulated H2A.X phosphorylation (Figure 2D), suggesting that galangin prevented PM_2.5_-induced DNA damage. Furthermore, we examined the effect of galangin on intracellular Ca^2+^ homeostasis using the calcium probe Fluo-4 AM. Galangin attenuated PM_2.5_-induced increase in intracellular Ca^2+^ level in HaCaT cells (Figure 2E). Additionally, we examined the mitochondrial membrane potential of the cells using JC-1 fluorescent probe and found that galangin ameliorated PM_2.5_-induced mitochondrial membrane depolarization (Figure 2F).

### Galangin protected against PM_2.5_-induced cell apoptosis

**Figure 3:**
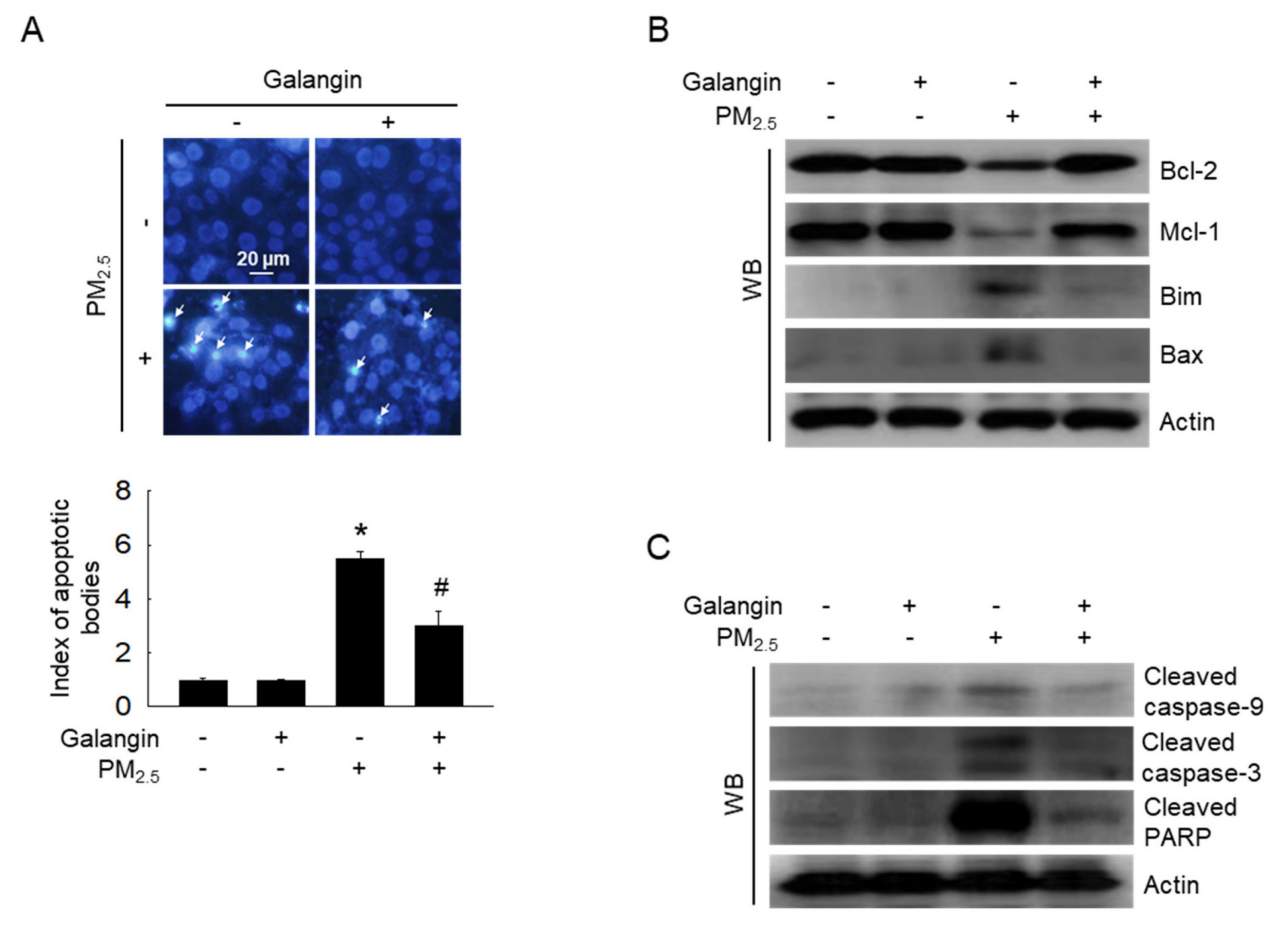
Inhibitory effect of galangin against PM_2.5_-induced cellular apoptosis in HaCaT cells. (A) Hoechst 33342 staining was used to identify the apoptotic bodies. Arrows mark their location. *p < 0.05, ^#^*p* < 0.05 versus control and PM_2.5_. (B) Protein levels of Bcl-2, Mcl-1, Bim, Bax (C) cleaved caspase-9, cleaved caspase-3, and cleaved PARP in HaCaT cell lysates were analyzed using western blotting, with actin as the loading control. Bcl-2, B-cell lymphoma 2; Mcl-1, myeloid cell leukemia-1; Bim, Bcl-2-like protein 11; Bax, Bcl-2-associated X protein; PARP, poly (ADP-ribose) polymerase.

Hoechst 33342 staining was performed to assess chromatin condensation, a well-known characteristic of apoptosis 21. PM_2.5_ exposure increased cell apoptosis, whereas galangin reversed these effects (*p* < 0.05; Figure 3A). Additionally, we examined the expression of apoptosis-associated proteins. PM_2.5_ exposure increased the expression of Bim, Bax, cleaved caspase-9, cleaved caspase-3, and cleaved PARP, and decreased the expression of Bcl-2 and Mcl-1 proteins (Figures 3B and 3C). However, galangin ameliorated PM_2.5_-induced changes in the expression of the apoptosis-related proteins, indicating that galangin can reduce cellular apoptosis.

### Galangin downregulated PM_2.5_-induced activation of the MAPK signaling pathway

**Figure 4:**
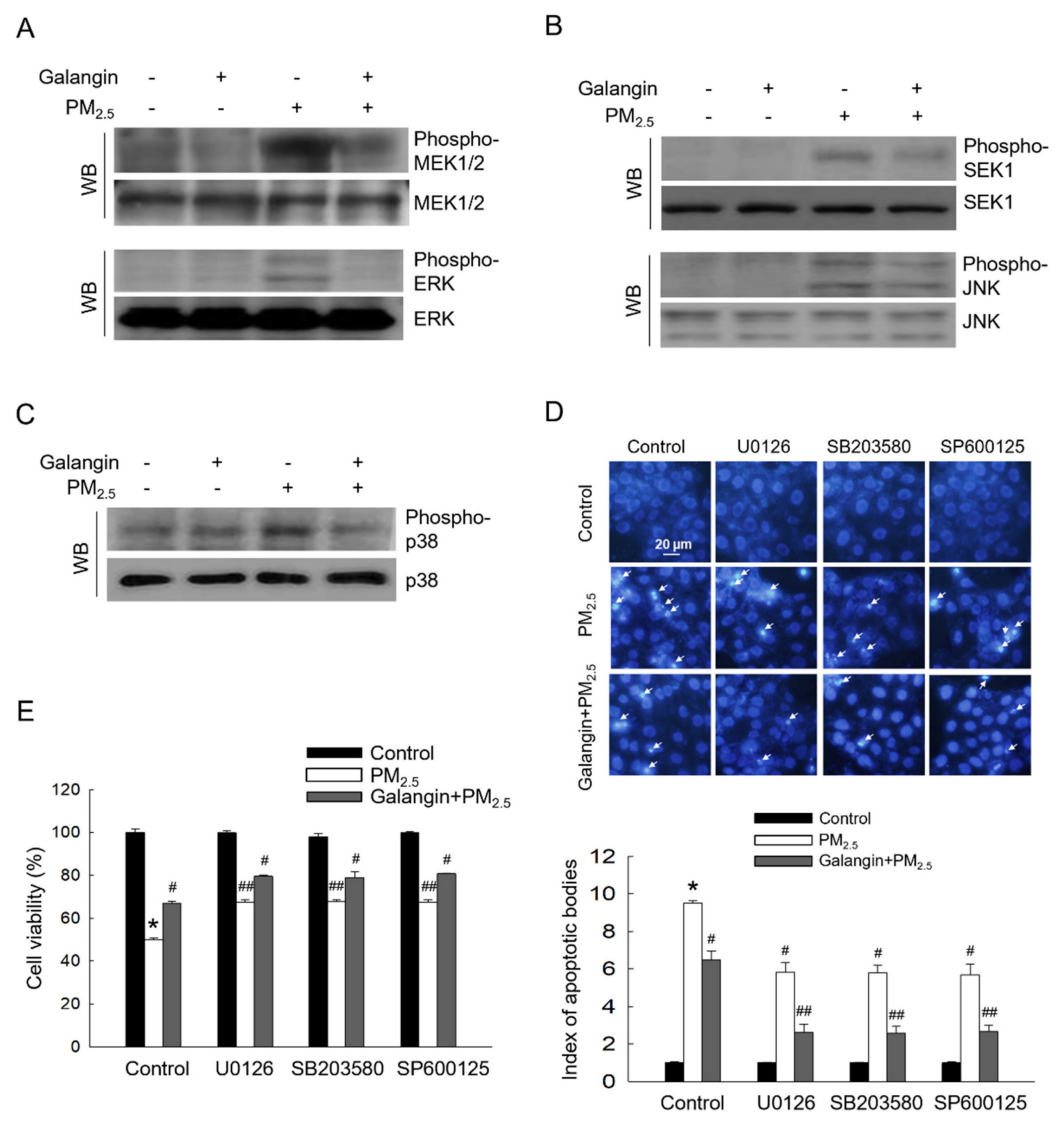
Downregulating effect of galangin against PM_2.5_-induced MAPK signaling pathway. (A) Phospho-MEK1/2, phospho-ERK, (B) phospho-SEK1, phospho-JNK, and (C) phospho-p38 in HaCaT cell lysates were analyzed using western blotting. MEK1/2, ERK, SEK1, JNK, and p38 were used as loading control. (D) Cell apoptosis level was analyzed using Hoechst 33342 staining, with arrows indicating apoptotic cells. (E) Cell viability was assessed using the MTT assay. (D, E) *p < 0.05, ^#^*p* < 0.05,^ ##^*p* < 0.05 versus control, PM_2.5_ and galangin+PM_2.5_. MEK1/2, mitogen-activated protein kinase kinase; ERK, extracellular signal-regulated kinase; SEK1, stress activated protein kinase (SAPK)/ERK kinase-1; JNK, c-Jun N-terminal kinase; PM_2.5_, particulate matter 2.5; MTT, 3-(4,5-dimethylthiazol-2-yl)-2,5-diphenyltetrazolium bromide.

PM_2.5_ exposure significantly increased MEK1/2, ERK, SEK1, JNK, and p38 phosphorylation, indicating activation of the MAPK signaling pathway. However, galangin attenuated the phosphorylation of these proteins (Figures 4A-4C). Importantly, treatment with ERK, p38, and JNK inhibitors (U0126, SB203580, and SP600125) attenuated PM_2.5_-induced cellular apoptosis and improved cell viability, suggesting that PM_2.5_ induces cell apoptosis by activating the MAPK signaling pathway (Figures 4D and 4E). Similarly, galangin mitigated PM_2.5_-induced cytotoxicity by inhibiting the MAPK signaling pathway (*p* < 0.05; Figures 4D and 4E).

### Galangin protected against PM_2.5_- and UVB-induced cellular cytotoxicity

**Figure 5:**
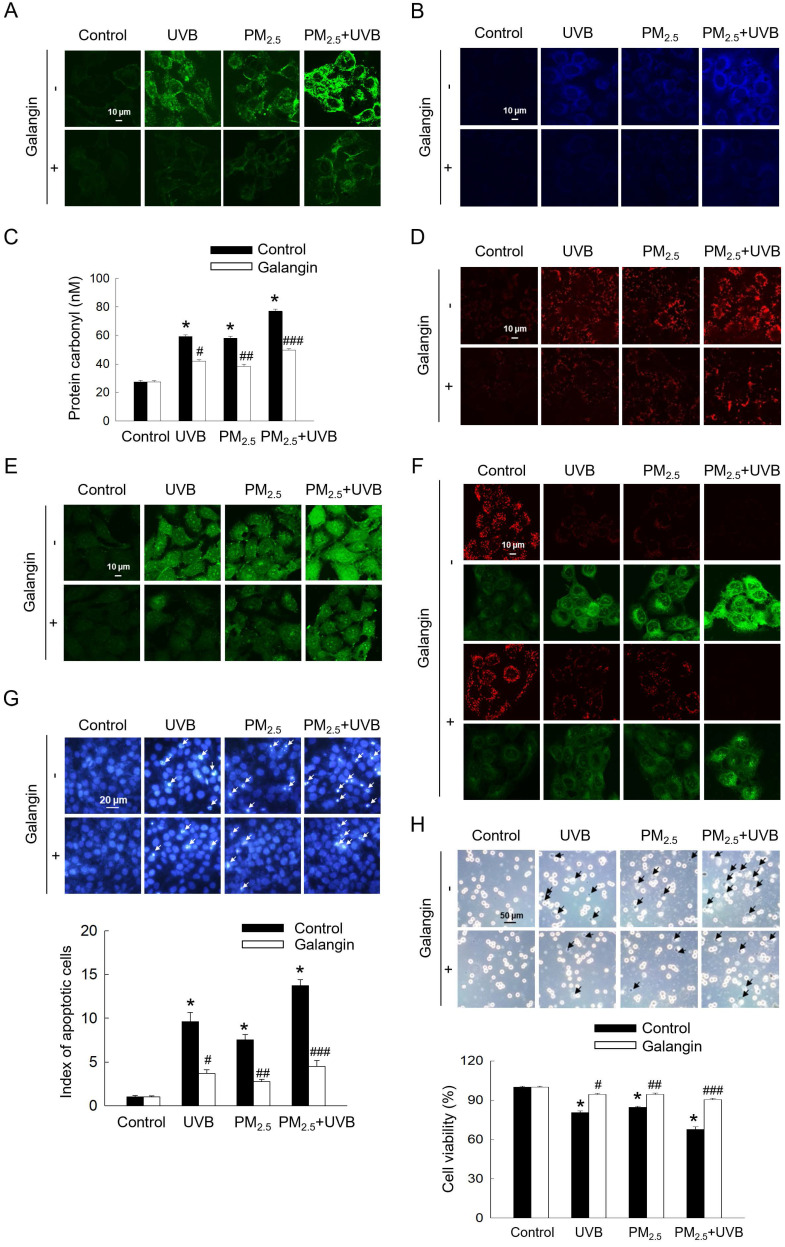
Protective effect of galangin against apoptosis induced by combined exposure to PM_2.5_ and UVB. (A) Intracellular ROS levels were evaluated using confocal microscopy after H_2_DCFDA staining. (B) Lipid peroxidation was observed using DPPP staining. (C) Protein carbonylation was assessed using a protein carbonylation assay. (D) 8-OxoG level was assessed using confocal microscopy after an avidin-TRITC conjugate staining. (E) Intracellular Ca^2+^ level was detected after Fluo-4 AM staining. (F) Mitochondrial membrane potential (Δψ_m_) was examined with JC-1 staining. (G) Apoptotic cells were observed after Hoechst 33342 staining. (H) Viable cells were detected using trypan blue staining. (C, G, H) *p < 0.05, ^#^*p* < 0.05,^ ##^*p* < 0.05,^ ###^*p* < 0.05 versus control, UVB, PM_2.5_ group and UVB+PM_2.5_. PM_2.5_, particulate matter 2.5; ROS, reactive oxygen species; H_2_DCFDA, 2′,7′-dichlorodihydrofluorescein diacetate; DPPP, diphenyl-1-pyrenylphosphine.

Considering that UVB exposure has been shown to induce oxidative stress and apoptosis in HaCaT cells 2, we investigated the aggravating effect of PM_2.5_ on UVB-induced keratinocyte damage. UVB and/or PM_2.5_ treatment enhanced intracellular ROS levels, lipid peroxidation, protein carbonylation, and 8-oxoG level, whereas galangin attenuated these cytotoxic effects (*p* < 0.05; Figures 5A-5D). Compared with PM_2.5_ treatment alone, PM_2.5_ and UVB co-exposure significantly increased intracellular Ca^2+^ level, as indicated by green fluorescence. However, galangin ameliorated the combined effect of PM_2.5_ and UVB on Ca^2+^ level (Figure 5E). Additionally, mitochondrial membrane depolarization was higher in the PM_2.5_+UVB group than in the UVB irradiation and PM_2.5_ treatment groups. Importantly, galangin reversed the combined effect of PM_2.5_ and UVB on mitochondrial membrane potential (Figure 5F). Notably, Hoechst 33342 staining and trypan blue assays revealed that PM_2.5_ treatment enhanced UVB-induced apoptosis and cell death. However, galangin provided partial protection against UVB and/or PM_2.5_-induced cell death (*p* < 0.05; Figures 5G and 5H). Collectively, these findings indicate that galangin exerts protective effects against UVB and/or PM_2.5_-induced oxidative damage and apoptosis.

## Discussion

The skin serves as a protective barrier that shields the body's internal organs from environmental threats, including physical, chemical, and biological agents, and thus helps maintain internal homeostasis 22. UV light, blue light, infrared light, and electromagnetic and sound waves are examples of physical pollutants, whereas PM_2.5_ and PAHs are chemical pollutants 23. PAHs are common constituents of diesel exhaust and lipophilic PAHs that permeate the skin and activate the AhR in keratinocytes, which increases cytochrome p450 expression and intracellular oxidative stress 24. Additionally, PAHs are recognized for their efficient ability to generate single oxygen via type-II photooxidation and act as photosensitising agents 22. Therefore, this study aimed to examine the protective effect of the polyphenol galangin against PM_2.5_- and UVB-induced oxidative damage.

Exposure to PM_2.5_ may increase intracellular ROS levels in HaCaT keratinocytes, leading to harmful effects 25, 26. Galangin has been shown to exert anti-oxidative effects against UVB-induced ROS production 2. Similarly, galangin mitigated PM_2.5_-induced ROS production, confirming its anti-oxidative effect. Oxidative stress caused by ROS is an important factor in cellular apoptosis. For example, oxidative stress-induced injury causes cellular macromolecular damage, mitochondrial damage, and apoptosis 27. During oxidative stress, ROS attack the polyunsaturated fatty acids in lipid membrane, causing lipid peroxidation. Lipid peroxidation increases cell membrane permeability, DNA mutations, and cell death 28. Protein carbonylation is a biomarker of oxidative stress resulting from protein damage 26. 8-OxoG and phospho-H2A.X are two molecular compounds that are distinct indicators of DNA damage and are upregulated in keratinocytes following PM_2.5_ exposure 29. Mitochondrial injury can lead to cellular degeneration, increased production of ROS, and energy depletion. Mitochondrial dysfunction, which is often caused by cellular stress, plays a key role in triggering apoptosis in a process involving abnormal expression of Bax and Bcl-2 proteins and the activation of caspases, particularly caspase-3 and -9 30. Notably, the pro-apoptotic and anti-apoptotic proteins Bax and Bcl-2 modulate the release of cytochrome c, which binds to apoptotic protease activating factor 1 in the cytoplasm and triggers the activation of caspase-9, leading to caspase-3 activation and a cascade of events that triggers apoptosis 2. In the present study, galangin reduced PM_2.5_-induced proapoptotic protein expression and restored anti-apoptotic protein levels.

The MAPK pathway comprises three key components: ERK, JNK, and p38. Cellular oxidative stress is associated with abnormal activation of MAPK pathways 30. Activation of the MAPK pathway is primarily associated with the promotion of apoptosis, especially in response to stress signals, such as UVB radiation and PM_2.5_ stress 31, 32. PM_2.5_ is believed to promote cellular apoptosis by activating p38 and JNK 33. The ERK pathway is involved in both cell survival and death. Recent studies have shown that prolonged ERK activation enhances cellular apoptosis 31. Our results demonstrate that PM_2.5_ exposure significantly increased the phosphorylation levels of ERK, JNK, and p38, verifying prior research that associated PM_2.5_ with MAPK activation and cellular death 26. Galangin reduced ERK, JNK, and p38 phosphorylation in groups exposed to PM_2.5_. This indicates that galangin may exert an inhibitory effect on environmental stress-induced MAPK signaling, possibly due to its antioxidant properties.

Although our study demonstrates the protective effects of galangin against PM_2.5_- and UVB-induced oxidative stress and apoptosis in HaCaT keratinocytes, the findings are based only on an *in vitro* model. Therefore, further *in vivo* studies are needed to confirm the protective efficacy and bioavailability of galangin under physiological conditions.

## Conclusions

Our findings reveal that PM_2.5_ aggravated skin cell damage by elevating ROS production and triggering apoptotic pathways. However, combined treatment with PM_2.5_ and UVB showed higher toxic effects than PM_2.5_ and UVB alone. Notably, galangin ameliorates these cytotoxic effects, demonstrating its protective properties against PM_2.5_- and UVB-induced damage in skin cells.

## Figures and Tables

**Figure 6:**
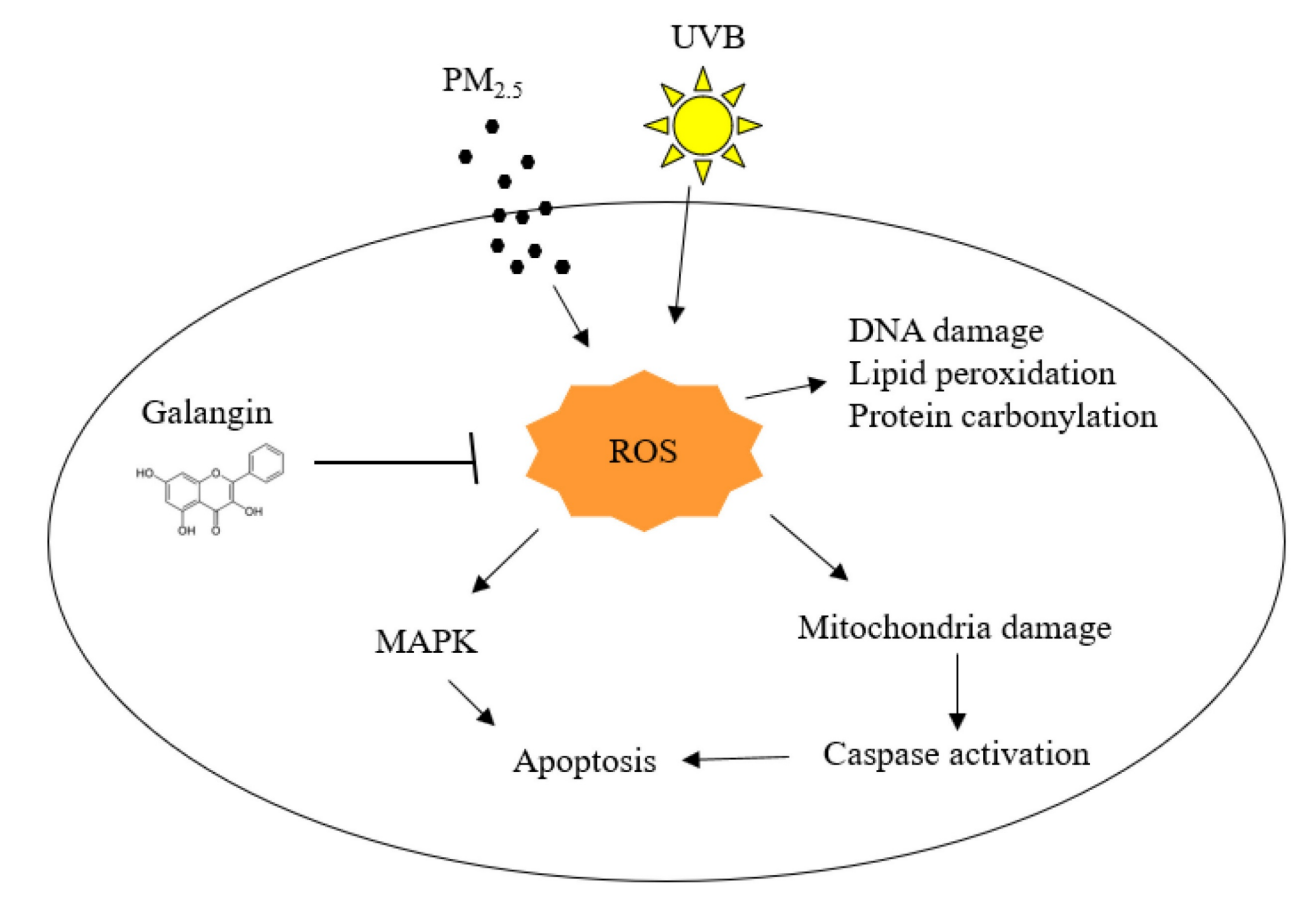
Effects of galangin on PM_2.5_- and UVB-induced keratinocyte damage. PM_2.5_ and UVB increased ROS production leading to oxidation of cellular components, MAPK activation, and mitochondria-mediated apoptosis, however, galangin ameliorated PM_2.5_- and UVB-induced cell damage via its ROS scavenging effect. PM_2.5_, particulate matter 2.5; UVB, ultraviolet B; ROS, reactive oxygen species; MAPK, mitogen-activated protein kinase.
